# 1,5-Bis(2-methyl­phen­yl)-3-nitro­formazan

**DOI:** 10.1107/S1600536812001171

**Published:** 2012-01-18

**Authors:** Karel G. von Eschwege, Eric C. Hosten, Alfred Muller

**Affiliations:** a Department of Chemistry, University of the Free State, PO Box 339, Bloemfontein 9300, South Africa; b Department of Chemistry, Nelson Mandela Metropolitan University, PO Box 77000, Port Elizabeth 6031, South Africa; c Research Center for Synthesis and Catalysis, Department of Chemistry, University of Johannesburg (APK Campus), PO Box 524, Auckland Park, Johannesburg 2006, South Africa

## Abstract

In the title compound, C_15_H_15_N_5_O_2_, the nitro O atoms are disordered over two sets of sites with an occupancy ratio of 0.75 (4):0.25 (4). Amine–imine tautomerism is observed in the formazan group. This was evident from the similar C—N bond distances in the formazan [1.319 (2) and 1.332 (3) Å], as well as the distribution of the imine proton in the Fourier difference map which refined to a 0.53 (3):0.47 (3) ratio. C—H⋯O and π–π inter­actions [centroid–centroid distances = 3.4813 (1) and 3.3976 (1) Å] are observed in the crystal packing.

## Related literature

For related structures of nitro­formazan derivatives, see: Gilroy *et al.* (2008 ▶); Mito *et al.* (1997 ▶) and for a related dithizone structure, see: Laing (1977 ▶). For the synthesis and chemistry of nitro­formazans, see: Pelkis *et al.* (1957 ▶); Irving (1977 ▶). For DFT and electrochemistry studies of dithizones, see: Von Eschwege & Swarts (2010 ▶); Von Eschwege *et al.* (2011 ▶).
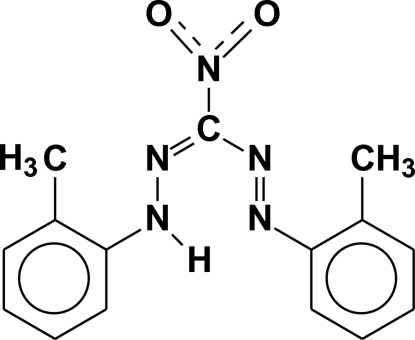


## Experimental

### 

#### Crystal data

C_15_H_15_N_5_O_2_
*M* *_r_* = 297.32
Orthorhombic, 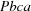

*a* = 14.6525 (3) Å
*b* = 10.2523 (3) Å
*c* = 19.2425 (4) Å
*V* = 2890.64 (12) Å^3^
*Z* = 8
Mo *K*α radiation
μ = 0.10 mm^−1^
*T* = 200 K
0.43 × 0.19 × 0.19 mm

#### Data collection

Bruker APEXII CCD diffractometer
Absorption correction: multi-scan (*SADABS*; Bruker, 2008 ▶) *T*_min_ = 0.960, *T*_max_ = 0.982
24031 measured reflections
3619 independent reflections
2487 reflections with *I* > 2σ(*I*)
*R*_int_ = 0.028

#### Refinement

*R*[*F* ^2^ > 2σ(*F* ^2^)] = 0.060
*wR*(*F* ^2^) = 0.180
*S* = 1.05
3619 reflections
221 parameters
48 restraints
H-atom parameters constrained
Δρ_max_ = 0.51 e Å^−3^
Δρ_min_ = −0.21 e Å^−3^

### 

Data collection: *APEX2* (Bruker, 2011 ▶); cell refinement: *SAINT* (Bruker, 2008 ▶); data reduction: *SAINT* and *XPREP* (Bruker, 2008 ▶); program(s) used to solve structure: *SIR97* (Altomare *et al.*, 1999 ▶); program(s) used to refine structure: *SHELXL97* (Sheldrick, 2008 ▶); molecular graphics: *DIAMOND* (Brandenburg & Putz, 2005 ▶); software used to prepare material for publication: *WinGX* (Farrugia, 1999 ▶).

## Supplementary Material

Crystal structure: contains datablock(s) global, I. DOI: 10.1107/S1600536812001171/kp2378sup1.cif

Structure factors: contains datablock(s) I. DOI: 10.1107/S1600536812001171/kp2378Isup2.hkl

Supplementary material file. DOI: 10.1107/S1600536812001171/kp2378Isup3.cml

Additional supplementary materials:  crystallographic information; 3D view; checkCIF report

## Figures and Tables

**Table 1:** Hydrogen-bond geometry (Å, °)

*D*—H⋯*A*	*D*—H	H⋯*A*	*D*⋯*A*	*D*—H⋯*A*
C4—H4⋯O1*A*^i^	0.95	2.42	3.239 (9)	145

Symmetry code: (i) 
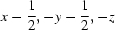
.

**Fig. 1.**
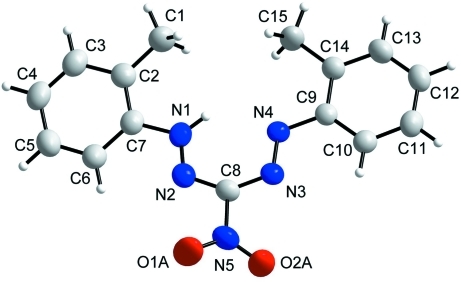
View of the title compound with labelling and displacement ellipsoids drawn at a 50% probability level. Hydrogen atoms and minor components of disorders not shown for clarity.

**Fig. 2.**
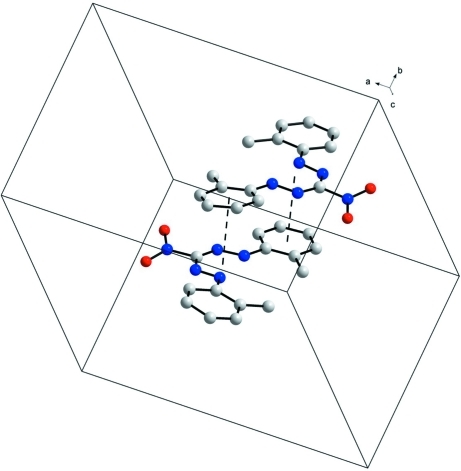
Partial packing diagram of title compound indicating the π···π interactions.

**Fig. 3.**
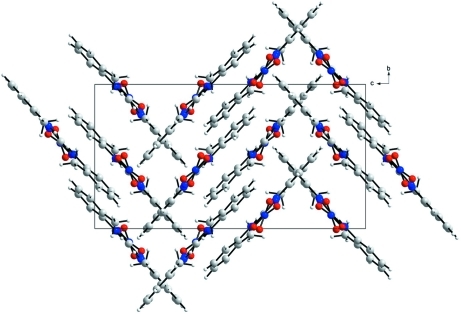
Packing diagram of the title compound viewed along the a axis illustrating the zigzag packing motif.

## supplementary crystallographic information

### Comment

During synthesis of the versatile trace metal analysis dithizone reagent, aniline is first diazotized and then treated with nitromethane to form the bright orange-red nitroformazan product (Pelkis *et al.*, 1957). Ammonia and hydrogen sulfide gas is used to substitute the nitro group with sulfur towards the formation of dithizone, the chemistry of which is extensively described by Irving, 1977. Single crystal X-ray structures of nitroformazan derivatives were determined by Gilroy *et al.*, 2008; Mito *et al.*,1997, and the dithizone structure by Laing, 1977, while we performed extensive DFT (Von Eschwege *et al.*, 2011) and electrochemistry studies (Von Eschwege & Swarts, 2010) on the free ligand. We recently embarked on a study during which we synthesized a series of electronically altered dithizones for the purpose of investigating its altered redox and structural properties. During this process orange 1,5-bis(2-methylphenyl)-3-nitroformazan crystals suitable for X-ray structure analysis were grown from an acetone solution overlaid with n-hexane.

The title compound (Fig. 1) crystallises with the 2-methylphenyl moieties in different orientations, *i.e.* C1—C7 and C9—C15 have dihedral angles of of 12.64 (9)° and 3.27 (9)° respectively with the N—N═ C—N═N backbone. The preferred orientations are probably due to the observed C—H···O interactions as well as the π-stacking of 2-methylphenyl aromatic rings (Tables 1 and 2, Fig. 2) with neighbouring N—N═C—N═N conjugates creating a zigzag packing motif (Fig. 3).

The structure showed large thermal vibrations at the NO_2_ moiety and was treated for disorder. From the Fourier difference map the imine hydrogen was also detected as being disordered over two nitrogen atoms. Details of these can be found in the refinement section.

### Experimental

Solvents (AR) purchased from Merck and reagents from Sigma-Aldrich were used without further purification. The *ortho*-methyl derivative of nitroformazan was prepared according to the procedure reported by Pelkis *et al.*, 1957.

### Refinement

All hydrogen atoms were positioned in geometrically idealized positions with 0.98 Å (methyl), 0.95 Å (aromatic) and C—H = 0.86 Å (imine). All hydrogen atoms were allowed to ride on their parent atoms with *U*_iso_(H) = 1.2*U*_eq_, except for the methyl where *U*_iso_(H) = 1.5*U*_eq_ was utilized. The initial positions of methyl hydrogen atoms were located from a Fourier difference map and refined as fixed rotor. The amine hydrogen atom was refined as disordered over N1 and N4. The occupancy was connected to a free variable to add to unity. This refined to a 0.53 (3):0.47 (3) ratio. The NO_2_ moiety showed large displacement ellipsoids on O1 and O2. These were also treated for disorder. Geometrical (SADI) and displacement (SIMU and DELU) restraints were applied. A free variable, to refine the disordered sites to unity, gave a distribution of 0.75 (4):0.25 (4).

### Crystal data

**Table:**

C_15_H_15_N_5_O_2_	*F*(000) = 1248
*M**_r_* = 297.32	*D*_x_ = 1.366 Mg m^−^^3^
Orthorhombic, *P**b**c**a*	Mo *K*α radiation, λ = 0.71073 Å
Hall symbol: -P 2ac 2ab	Cell parameters from 8384 reflections
*a* = 14.6525 (3) Å	θ = 2.7–28.3°
*b* = 10.2523 (3) Å	µ = 0.10 mm^−^^1^
*c* = 19.2425 (4) Å	*T* = 200 K
*V* = 2890.64 (12) Å^3^	Needle, red
*Z* = 8	0.43 × 0.19 × 0.19 mm

### Data collection

**Table:**

Bruker APEXII CCD diffractometer	3619 independent reflections
graphite	2487 reflections with *I* > 2σ(*I*)
Detector resolution: 8.4 pixels mm^-1^	*R*_int_ = 0.028
ω and φ scans	θ_max_ = 28.4°, θ_min_ = 2.1°
Absorption correction: multi-scan (*SADABS*; Bruker, 2008)	*h* = −19→19
*T*_min_ = 0.960, *T*_max_ = 0.982	*k* = −12→13
24031 measured reflections	*l* = −25→25

### Refinement

**Table:**

Refinement on *F*^2^	Primary atom site location: structure-invariant direct methods
Least-squares matrix: full	Secondary atom site location: difference Fourier map
*R*[*F*^2^ > 2σ(*F*^2^)] = 0.060	Hydrogen site location: inferred from neighbouring sites
*wR*(*F*^2^) = 0.180	H-atom parameters constrained
*S* = 1.05	*w* = 1/[σ^2^(*F*_o_^2^) + (0.070*P*)^2^ + 1.8629*P*] where *P* = (*F*_o_^2^ + 2*F*_c_^2^)/3
3619 reflections	(Δ/σ)_max_ < 0.001
221 parameters	Δρ_max_ = 0.51 e Å^−^^3^
48 restraints	Δρ_min_ = −0.21 e Å^−^^3^

### Special details

**Table:**

Experimental. The intensity data was collected on a Bruker *APEX*-II CCD diffractometer using an exposure time of 60 s/frame. A total of 1062 frames were collected with a frame width of 0.5° covering up to θ = 28.40° with 99.6% completeness accomplished.Analytical data: *M*.p. 154 °C. λ_max_ (dichloromethane) 319, 440 nm. ^1^H (600 MHz, CDCl_3_) 14.32 (1 H, 1 × s, 1 × NH), 2.58 (6 H, 1 × s, 2 × CH_3_), 7.92 – 7.29 (8 H, 2 × m, 2 × C_6_H_4_)
Geometry. All e.s.d.'s (except the e.s.d. in the dihedral angle between two l.s. planes) are estimated using the full covariance matrix. The cell e.s.d.'s are taken into account individually in the estimation of e.s.d.'s in distances, angles and torsion angles; correlations between e.s.d.'s in cell parameters are only used when they are defined by crystal symmetry. An approximate (isotropic) treatment of cell e.s.d.'s is used for estimating e.s.d.'s involving l.s. planes.
Refinement. Refinement of *F*^2^ against ALL reflections. The weighted *R*-factor *wR* and goodness of fit *S* are based on *F*^2^, conventional *R*-factors *R* are based on *F*, with *F* set to zero for negative *F*^2^. The threshold expression of *F*^2^ > σ(*F*^2^) is used only for calculating *R*-factors(gt) *etc*. and is not relevant to the choice of reflections for refinement. *R*-factors based on *F*^2^ are statistically about twice as large as those based on *F*, and *R*- factors based on ALL data will be even larger.

### Fractional atomic coordinates and isotropic or equivalent isotropic displacement parameters (Å^2^)

**Table:**

	*x*	*y*	*z*	*U*_iso_*/*U*_eq_	Occ. (<1)
N1	0.26169 (12)	0.01378 (16)	0.07679 (8)	0.0441 (4)	
HN1	0.2252	0.0663	0.0999	0.053*	0.53 (3)
N2	0.34854 (12)	0.01558 (17)	0.08281 (8)	0.0458 (4)	
N3	0.35427 (11)	0.19544 (16)	0.16744 (8)	0.0408 (4)	
N4	0.26631 (10)	0.21162 (16)	0.17126 (8)	0.0385 (4)	
HN4	0.2283	0.1648	0.1462	0.046*	0.47 (3)
N5	0.48561 (13)	0.0936 (2)	0.12737 (11)	0.0580 (5)	
O1A	0.5222 (5)	−0.0056 (8)	0.1083 (9)	0.094 (3)	0.75 (4)
O2A	0.5302 (5)	0.1873 (10)	0.1454 (10)	0.087 (3)	0.75 (4)
O1B	0.512 (2)	0.012 (3)	0.087 (2)	0.107 (8)	0.25 (4)
O2B	0.5218 (19)	0.155 (4)	0.172 (2)	0.097 (6)	0.25 (4)
C1	0.07297 (15)	−0.0431 (3)	0.07535 (13)	0.0596 (6)	
H1A	0.092	−0.0557	0.1237	0.089*	
H1B	0.0118	−0.0797	0.0687	0.089*	
H1C	0.072	0.0503	0.0646	0.089*	
C2	0.13763 (15)	−0.1095 (2)	0.02891 (10)	0.0485 (5)	
C3	0.10754 (17)	−0.2092 (2)	−0.01616 (11)	0.0549 (6)	
H3	0.0448	−0.2327	−0.0162	0.066*	
C4	0.16569 (18)	−0.2723 (2)	−0.05938 (12)	0.0590 (6)	
H4	0.1436	−0.3392	−0.0891	0.071*	
C5	0.25748 (19)	−0.2394 (2)	−0.06022 (12)	0.0629 (6)	
H5	0.2978	−0.2822	−0.0915	0.075*	
C6	0.28997 (16)	−0.1465 (2)	−0.01666 (11)	0.0543 (5)	
H6	0.353	−0.1251	−0.0169	0.065*	
C7	0.22993 (15)	−0.0819 (2)	0.02888 (10)	0.0464 (5)	
C8	0.38492 (13)	0.1033 (2)	0.12568 (9)	0.0425 (4)	
C9	0.23522 (12)	0.30827 (18)	0.21773 (9)	0.0375 (4)	
C10	0.29585 (14)	0.3870 (2)	0.25478 (10)	0.0448 (5)	
H10	0.3598	0.3751	0.2496	0.054*	
C11	0.26305 (16)	0.4823 (2)	0.29902 (10)	0.0501 (5)	
H11	0.3043	0.5367	0.3238	0.06*	
C12	0.16987 (16)	0.4981 (2)	0.30707 (11)	0.0510 (5)	
H12	0.1469	0.5632	0.3375	0.061*	
C13	0.11046 (15)	0.4192 (2)	0.27091 (11)	0.0511 (5)	
H13	0.0467	0.431	0.2771	0.061*	
C14	0.14087 (13)	0.3228 (2)	0.22551 (10)	0.0429 (4)	
C15	0.07500 (14)	0.2388 (3)	0.18674 (14)	0.0619 (6)	
H15A	0.0125	0.2619	0.2001	0.093*	
H15B	0.0864	0.147	0.198	0.093*	
H15C	0.0829	0.2523	0.1367	0.093*	

### Atomic displacement parameters (Å^2^)

**Table:**

	*U*^11^	*U*^22^	*U*^33^	*U*^12^	*U*^13^	*U*^23^
N1	0.0552 (10)	0.0391 (9)	0.0381 (8)	−0.0027 (7)	−0.0009 (7)	0.0037 (7)
N2	0.0527 (9)	0.0440 (9)	0.0405 (8)	0.0019 (8)	0.0020 (7)	0.0042 (7)
N3	0.0402 (8)	0.0431 (9)	0.0392 (8)	0.0031 (7)	0.0009 (6)	0.0042 (7)
N4	0.0381 (8)	0.0405 (9)	0.0369 (7)	0.0013 (6)	0.0012 (6)	0.0042 (6)
N5	0.0436 (10)	0.0637 (13)	0.0666 (12)	0.0095 (9)	0.0059 (9)	−0.0043 (10)
O1A	0.045 (2)	0.070 (3)	0.168 (7)	0.0139 (19)	0.001 (3)	−0.030 (3)
O2A	0.0410 (16)	0.094 (4)	0.125 (6)	−0.0035 (18)	−0.004 (3)	−0.048 (4)
O1B	0.085 (11)	0.124 (13)	0.112 (14)	0.049 (10)	0.039 (9)	−0.025 (9)
O2B	0.049 (7)	0.141 (14)	0.101 (12)	−0.003 (8)	−0.011 (8)	−0.027 (11)
C1	0.0484 (12)	0.0678 (15)	0.0626 (13)	−0.0005 (11)	−0.0058 (10)	−0.0057 (12)
C2	0.0552 (12)	0.0461 (12)	0.0440 (10)	0.0007 (9)	0.0014 (9)	0.0074 (9)
C3	0.0651 (13)	0.0518 (13)	0.0479 (11)	−0.0098 (11)	−0.0066 (10)	0.0042 (10)
C4	0.0763 (15)	0.0480 (13)	0.0528 (12)	−0.0119 (11)	−0.0020 (11)	−0.0008 (10)
C5	0.0755 (16)	0.0577 (14)	0.0555 (13)	−0.0040 (12)	0.0140 (12)	−0.0069 (11)
C6	0.0601 (13)	0.0548 (13)	0.0481 (11)	−0.0088 (10)	0.0075 (9)	−0.0021 (10)
C7	0.0567 (12)	0.0431 (11)	0.0394 (9)	−0.0034 (9)	−0.0013 (8)	0.0056 (8)
C8	0.0400 (9)	0.0454 (11)	0.0420 (9)	0.0042 (8)	0.0020 (8)	0.0043 (8)
C9	0.0431 (9)	0.0360 (9)	0.0334 (8)	0.0018 (7)	0.0008 (7)	0.0066 (7)
C10	0.0465 (10)	0.0472 (11)	0.0407 (9)	−0.0020 (9)	−0.0003 (8)	0.0031 (8)
C11	0.0625 (13)	0.0466 (12)	0.0413 (10)	−0.0026 (10)	−0.0034 (9)	0.0009 (9)
C12	0.0644 (13)	0.0438 (12)	0.0449 (11)	0.0059 (10)	0.0036 (9)	−0.0019 (9)
C13	0.0495 (11)	0.0474 (12)	0.0564 (12)	0.0082 (9)	0.0065 (9)	0.0001 (10)
C14	0.0438 (10)	0.0404 (10)	0.0445 (10)	0.0035 (8)	0.0008 (8)	0.0029 (8)
C15	0.0408 (10)	0.0639 (15)	0.0811 (16)	0.0020 (10)	−0.0016 (10)	−0.0208 (13)

### Geometric parameters (Å, °)

**Table:**

N1—N2	1.278 (2)	C4—C5	1.387 (4)
N1—C7	1.424 (3)	C4—H4	0.95
N1—HN1	0.88	C5—C6	1.355 (3)
N2—C8	1.332 (3)	C5—H5	0.95
N3—N4	1.302 (2)	C6—C7	1.407 (3)
N3—C8	1.319 (2)	C6—H6	0.95
N4—C9	1.410 (2)	C9—C10	1.396 (3)
N4—HN4	0.88	C9—C14	1.399 (3)
N5—O2B	1.192 (13)	C10—C11	1.382 (3)
N5—O1A	1.208 (5)	C10—H10	0.95
N5—O1B	1.210 (13)	C11—C12	1.384 (3)
N5—O2A	1.212 (6)	C11—H11	0.95
N5—C8	1.479 (3)	C12—C13	1.377 (3)
C1—C2	1.470 (3)	C12—H12	0.95
C1—H1A	0.98	C13—C14	1.392 (3)
C1—H1B	0.98	C13—H13	0.95
C1—H1C	0.98	C14—C15	1.494 (3)
C2—C7	1.382 (3)	C15—H15A	0.98
C2—C3	1.411 (3)	C15—H15B	0.98
C3—C4	1.355 (3)	C15—H15C	0.98
C3—H3	0.95		
			
N2—N1—C7	113.21 (17)	C5—C6—C7	119.8 (2)
N2—N1—HN1	123.4	C5—C6—H6	120.1
C7—N1—HN1	123.4	C7—C6—H6	120.1
N1—N2—C8	117.68 (17)	C2—C7—C6	121.0 (2)
N4—N3—C8	117.57 (16)	C2—C7—N1	117.44 (19)
N3—N4—C9	116.44 (15)	C6—C7—N1	121.53 (19)
N3—N4—HN4	121.8	N3—C8—N2	136.45 (18)
C9—N4—HN4	121.8	N3—C8—N5	111.97 (17)
O2B—N5—O1A	117.8 (14)	N2—C8—N5	111.57 (17)
O2B—N5—O1B	134 (2)	C10—C9—C14	120.83 (18)
O1A—N5—O2A	121.0 (5)	C10—C9—N4	121.64 (17)
O1B—N5—O2A	124.2 (14)	C14—C9—N4	117.53 (16)
O2B—N5—C8	115.1 (14)	C11—C10—C9	120.14 (19)
O1A—N5—C8	119.5 (4)	C11—C10—H10	119.9
O1B—N5—C8	110.4 (16)	C9—C10—H10	119.9
O2A—N5—C8	119.4 (4)	C10—C11—C12	119.6 (2)
C2—C1—H1A	109.5	C10—C11—H11	120.2
C2—C1—H1B	109.5	C12—C11—H11	120.2
H1A—C1—H1B	109.5	C13—C12—C11	119.9 (2)
C2—C1—H1C	109.5	C13—C12—H12	120
H1A—C1—H1C	109.5	C11—C12—H12	120
H1B—C1—H1C	109.5	C12—C13—C14	122.1 (2)
C7—C2—C3	117.0 (2)	C12—C13—H13	118.9
C7—C2—C1	122.4 (2)	C14—C13—H13	118.9
C3—C2—C1	120.5 (2)	C13—C14—C9	117.34 (18)
C4—C3—C2	121.8 (2)	C13—C14—C15	121.07 (19)
C4—C3—H3	119.1	C9—C14—C15	121.59 (18)
C2—C3—H3	119.1	C14—C15—H15A	109.5
C3—C4—C5	120.0 (2)	C14—C15—H15B	109.5
C3—C4—H4	120	H15A—C15—H15B	109.5
C5—C4—H4	120	C14—C15—H15C	109.5
C6—C5—C4	120.3 (2)	H15A—C15—H15C	109.5
C6—C5—H5	119.8	H15B—C15—H15C	109.5
C4—C5—H5	119.8		
			
C7—N1—N2—C8	178.96 (16)	O1A—N5—C8—N3	159.5 (10)
C8—N3—N4—C9	178.28 (15)	O1B—N5—C8—N3	−177 (2)
C7—C2—C3—C4	−2.2 (3)	O2A—N5—C8—N3	−23.3 (11)
C1—C2—C3—C4	−179.8 (2)	O2B—N5—C8—N2	−169 (3)
C2—C3—C4—C5	−0.1 (4)	O1A—N5—C8—N2	−19.9 (10)
C3—C4—C5—C6	1.7 (4)	O1B—N5—C8—N2	3(2)
C4—C5—C6—C7	−0.9 (4)	O2A—N5—C8—N2	157.4 (11)
C3—C2—C7—C6	3.0 (3)	N3—N4—C9—C10	3.3 (2)
C1—C2—C7—C6	−179.5 (2)	N3—N4—C9—C14	−176.70 (16)
C3—C2—C7—N1	−176.77 (17)	C14—C9—C10—C11	−1.0 (3)
C1—C2—C7—N1	0.8 (3)	N4—C9—C10—C11	178.91 (17)
C5—C6—C7—C2	−1.5 (3)	C9—C10—C11—C12	0.8 (3)
C5—C6—C7—N1	178.3 (2)	C10—C11—C12—C13	−0.2 (3)
N2—N1—C7—C2	167.38 (17)	C11—C12—C13—C14	−0.3 (3)
N2—N1—C7—C6	−12.4 (3)	C12—C13—C14—C9	0.1 (3)
N4—N3—C8—N2	0.3 (3)	C12—C13—C14—C15	−179.8 (2)
N4—N3—C8—N5	−178.83 (16)	C10—C9—C14—C13	0.6 (3)
N1—N2—C8—N3	0.8 (3)	N4—C9—C14—C13	−179.37 (16)
N1—N2—C8—N5	179.90 (16)	C10—C9—C14—C15	−179.5 (2)
O2B—N5—C8—N3	11 (3)	N4—C9—C14—C15	0.5 (3)

### Hydrogen-bond geometry (Å, °)

**Table:**

*D*—H···*A*	*D*—H	H···*A*	*D*···*A*	*D*—H···*A*
C4—H4···O1A^i^	0.95	2.42	3.239 (9)	145

Symmetry codes: (i) *x*−1/2, −*y*−1/2, −*z*.

### Table 2 Table 2. Short π···π interaction geometries (°, Å)

**Table:**

*Cg*(X)···*Cg*(Y)	*Cg*···*Cg*	Alpha	Beta	Gamma	*Cg*(X)perp	*Cg*(X)perp
*Cg*1···*Cg*2^i^	3.4813	7.215	4.85	12.04	3.4047	-3.4688
*Cg*1···*Cg*3^i^	3.3976	2.589	3.11	3.37	-3.3917	3.3925

For centroids: *Cg*1 = N_1_—N_2_═C_8_—N_3_═N_4_, *Cg*2 = ring C_2_ – C_7_, *Cg*3 = ring C_9_ – C_14_; Symmetry codes: i = 1/2-X,1/2+Y,Z; Alpha = Dihedral angle between *Cg*(X) and *Cg*(Y); *Cg*(X)perp = Perpendicular distance of *Cg*(X) on ring Y; *Cg*(X)perp = Perpendicular distance of *Cg*(Y) on ring X; Beta = Angle *Cg*(X)···*Cg*(Y) vector and normal to ring X; Gamma = Angle *Cg*(I)···*Cg*(J) vector and normal to plane Y;
